# Achieving ACGME Clinical Informatics Fellowship Accreditation: A Chronicle of Our Institution's Journey

**DOI:** 10.1055/a-2680-5920

**Published:** 2025-09-26

**Authors:** Srikar Chamala, Matthew S. Keefer, Troy McGuire, Leticia Diaz, Anthony A. Luberti, Christoph U. Lehmann, Ryan J. Schmidt

**Affiliations:** 1 Department of Pathology and Laboratory Medicine, Children's Hospital Los Angeles, California, United States; 2 Department of Pathology, Keck School of Medicine, University of Southern California, California, United States; 3 Department of General Pediatrics, Children's Hospital Los Angeles, Los Angeles, California, United States; 4 Department of Biomedical and Health Informatics, The Children's Hospital of Philadelphia, Philadelphia, Pennsylvania, United States; 5 Clinical Informatics Center, University of Texas Southwestern Medical Center, Dallas, Texas, United States; 6 Department of Pediatrics, University of Texas Southwestern Medical Center, Dallas, Texas, United States

**Keywords:** clinical informatics, fellowship, ACGME accreditation, graduate medical education, physician, workforce, ACGME, medical informatics, CHLA

## Abstract

**Background:**

Clinical informatics (CI) is a dynamic field at the intersection of health care, technology, and information science, aimed at enhancing health care delivery and patient outcomes. CI fellowship programs prepare physicians to lead and apply health information technologies that drive improvements in clinical care, system efficiency, and innovation. Achieving Accreditation Council for Graduate Medical Education (ACGME) accreditation for such programs is crucial for standardizing training and ensuring the development of a competent workforce.

**Objectives:**

This study aims to share our institution's experience at Children's Hospital Los Angeles in obtaining ACGME accreditation for a CI fellowship program, highlighting key steps and strategies.

**Methods:**

A multidisciplinary steering committee, including institutional leaders and CI experts, was formed. Key activities included a needs assessment, curriculum development, securing institutional support, and engaging with external advisors from accredited CI programs. The curriculum was designed to align with ACGME core competencies and the American Board of Preventive Medicine's CI subspecialty requirements. Extensive documentation, including rotation schedules and evaluation forms, was prepared for submission.

**Results:**

Our CI fellowship program received ACGME accreditation. Essential components that contributed to our success included strong institutional support, a well-defined curriculum integrating clinical and non-clinical rotations, and strategic faculty involvement across various informatics domains. Guidance from experienced directors at established CI programs was instrumental in expediting our application process and ensuring program readiness. The development of detailed documentation, such as a block diagram and competency-based evaluations, was crucial in demonstrating compliance with ACGME requirements.

**Conclusion:**

Our experience of achieving ACGME accreditation underscores the importance of assembling a diverse steering committee, engaging external expertise, and securing robust faculty participation. The strategies and insights gained from our accreditation journey can serve as a roadmap for other institutions aiming to establish CI fellowship programs, which are vital for training future leaders in CI and advancing health care innovation.

## Background and Significance

Clinical informatics (CI) is an interdisciplinary, rapidly changing field at the intersection of health care science, information science and technology, and social/behavioral science. 1 CI uses data, information, and knowledge to enhance the delivery of health care services. More recently, this includes the applications of machine learning and artificial intelligence in health care, which have the potential to revolutionize medical practice.

The formal recognition of CI as a medical subspecialty and its ACGME milestones are built upon the pioneering efforts of several key leaders in the field. Notably, Dr. Don Detmer, Dr. Ted Shortliffe, Dr. Chris Lehmann, Dr. Peter Elkin, and many others played instrumental roles in establishing the academic and structural foundations of CI as a formal medical subspecialty and continue to influence its evolution. 2 3 4 5 6 The purpose of CI fellowship programs is to equip physicians with proficiency in the application, implementation, and management of health care informatics technologies, addressing the challenges and opportunities for medical practice and research. Successfully completing a CI fellowship allows fellows to become board-eligible. 6 7 8 9 Unlike traditional ACGME-accredited fellowships—which typically focus on a single clinical subspecialty within a defined department—CI fellowships are inherently interdisciplinary. Fellows engage with a broad range of stakeholders, including clinicians, data scientists, software developers, and IT leaders, spanning multiple domains of health care. This cross-functional training fosters systems-level thinking and prepares fellows to lead informatics initiatives across the entire health care enterprise. These features make CI fellowships distinct within the landscape of ACGME-accredited fellowships.

The unique characteristics of the CI fellowship program outlined above present distinct challenges that require thorough planning, strong institutional support, and active engagement from a diverse group of stakeholders to ensure successful implementation. Our institution embarked on a mission to obtain ACGME accreditation with the aim of training future leaders in CI. This manuscript offers a detailed account of our journey and shares key experiences, insights, and lessons learned throughout the process of obtaining ACGME accreditation for the CI fellowship program at Children's Hospital Los Angeles (CHLA).

## Program Development Process

### Institutional Clinical Informatics Fellowship Steering Committee and External Advice for ACGME Accreditation

The formation of an institutional CI Fellowship Steering Committee for ACGME Accreditation was a pivotal first step in the journey toward successful application for ACGME accreditation of our fellowship program. The committee included a diverse group of stakeholders, including departmental and institutional leaders responsible for financial support, leading experts in CI at our institution, and faculty candidates eligible for the Program Director and Associate Director roles (board-certified in CI). Integral members aiding in financial support included the sponsoring Department Chair and Administrative Director.

The core committee—consisting of the Chief Medical Information Officer (MD, CI Board-Certified), Chief Health Information Officer (MD, CI Board-Certified), prospective CI Fellowship Program Director (MD, PhD, CI Board-Certified), Associate Program Director (PhD), and the Program Coordinator (MEd)—met every 2 weeks to develop and implement a strategic roadmap, addressing key milestones at each step.

Among the committee members, three are board-certified in CI through the practice pathway. While the Associate Program Director does not hold an MD or clinical board certification, he brings deep expertise in biomedical informatics, CI operations, and data science. He previously served as Director of Biomedical Informatics at his former institution and currently leads Pathology Informatics and Data Science at the present institution. He has also led the development of multiple informatics training curricula for medical trainees, several of which have been published. 10 11 12 13 Additionally, our Program Coordinator brings substantial experience in coordinating medical fellowship programs and contributes valuable administrative leadership to the team.

This committee's varied expertise was crucial in our thorough planning and implementation. The composition of such a committee will depend on the specific organizational structure and power dynamics of an institution. Ideally, during the planning phase for securing CI fellowship accreditation, the committee should consist of leaders and faculty who can secure funding to cover the cost of the CI fellowship program, comprehend the breadth of enterprise CI (e.g., Chief Medical Information Officer [CMIO]), and identify and enlist faculty for the program. It is imperative to include members who are CI board-certified and have experience in well-established CI programs. Additionally, incorporating a liaison to the local Graduate Medical Education (GME) office, along with a strong relationship with this office, and a thorough familiarity with ACGME-accreditation procedures, are essential.

In addition to forming our institutional steering committee, we actively pursued and received extensive guidance from program directors at external institutions with well-established CI fellowship programs. This collaborative effort was invaluable, expediting our application process and bolstering the likelihood of a successful program launch by providing access to expert advice and examples of previously successful application materials. Notably, Dr. Anthony A. Luberti, Program Director of The Children's Hospital of Philadelphia CI fellowship program, and Dr. Christoph U. Lehmann, former Program Director at the University of Texas Southwestern and the Vanderbilt University Medical Center CI fellowship programs, generously guided us through our CI fellowship application process and are coauthors of this manuscript.

### Identifying Prospective Faculty for Clinical Informatics Fellowship

The core members of the CI Fellowship Steering Committee (listed above) were responsible for guiding program design and implementation, including the identification and recruitment of qualified faculty.

Given the interdisciplinary nature of CI, a successful training program must engage faculty across multiple medical informatics specialties. Thus, the program needs to work across the traditional departmental boundaries that define academic medicine. CI teaching faculty should have expertise in informatics within their area of the medical enterprise. The faculty may or may not be board-certified in CI. In fact, it is valuable to expose CI fellows to faculty who may not have medical or academic backgrounds but hold pivotal executive or administrative positions in CI. Notable positions include the Chief Information Officer, Chief Data Officer, and the Chief Information Security Officer, whose insights and experiences are invaluable to a comprehensive educational experience.

The fellowship steering committee first identified rotation topics and mapped potential qualified candidates based on their expertise and academic achievements within our institution, who could serve as faculty for each topic. In some cases, the committee assigned multiple candidates to a given rotation topic based on the complexity of the domain and availability of faculty. After completing this task, the committee contacted faculty to confirm their interest and commitment to participate in training CI fellows. Our experience was incredibly positive: 100% of the faculty contacted expressed great enthusiasm to support the program and to participate as a faculty member. While the ACGME application process does not mandate official commitment letters from faculty at the application stage, it does require documentation of qualifications. The committee gathered supporting materials, including informatics experience, publications, presentations, and grant histories, to confirm eligibility for CI faculty roles.

For our CI program, faculty play a pivotal role in designing the curriculum for their respective rotations, including didactic lectures and practical, hands-on training. Many also pair fellows with their staff for shadowing opportunities and work on projects of interest to the trainee and department. This approach not only enriches the fellows' experience but also distributes the teaching workload more effectively by sharing the training responsibilities with their staff while ensuring that the quality of the fellows' experiences remains high.

For program governance, one faculty member must be appointed as the Program Director. This individual is endowed with authority over and is accountable for the entire program and must ensure adherence to all program requirements. The Program Director must be board-certified in CI. Additionally, there may be one or more faculty members serving as Associate Program Directors, who do not have to be a physician (Doctor of Medicine or Doctor of Osteopathic Medicine) and who do not have to be board certified in CI.

The Program Director was chosen based on a combination of qualifications, including board certification in CI, and a commitment to dedicate at least 20% of professional effort to fellowship activities. Importantly, the selected individual had also expressed a strong interest in mentoring and advancing educational initiatives within the institution. This decision was made collaboratively by the steering committee to ensure alignment with both ACGME requirements and our institutional goals for the fellowship.

### Program Design Roadmap

With faculty identified, the core members of the CI Fellowship Steering Committee—working closely with CI faculty—shifted from high-level planning to the detailed design of the program. This phase involved structuring rotations, selecting instructional formats, and aligning the curriculum with ACGME requirements.

The development process is outlined in Table 1 , which presents the key planning and implementation milestones that shaped the fellowship's progression from initial concept to final design.

**Table 1:** Key steps in the planning, preparation, and approval process for our ACGME Clinical Informatics Fellowship

Key activity and description	Timeline
Step 1: Establish Clinical Informatics Fellowship Steering Committee • Identified members and established the Clinical Informatics Fellowship Steering Committee • Met every 2 weeks to develop and follow a strategic roadmap, addressing key milestones at each stage. • Sought and received guidance from program directors at external institutions with well-established clinical informatics fellowship programs to support the ACGME-accreditation process.	Month 1
Step 2: Confirmed Faculty and Rotations • Identified and confirmed all clinical informatics fellowship teaching faculty, including both core and non-core members. This includes all faculty who will be listed on the ACGME roster for this fellowship. • Obtained CVs for all faculty members who will be on the roster. Information from these CVs was entered into the Accreditation Data System roster. • Discussed and finalized rotation topics and didactics. ADS: A web-based system that houses essential accreditation data for all Sponsoring Institutions and Programs within the ACGME. This is the platform where the applications will be submitted.	Months 2-7
Step 3: Prepared documents for the ACGME CI application (Refer to “Key Submission Documents” section for more details) • Block Diagram • Program Letters of Agreement (as needed) • Policy for Clinical and Educational Work Hours • Policy for Supervision of Fellows • Policy for Fellow and Faculty Member Well-Being • Rotation-Specific Goals and Objectives for all proposed rotations (refer to Appendix S1 [available in the online version only] for a sample) • Required by the CHLA Graduate Medical Education Office for review, not the ACGME • Semiannual and Final Evaluation of Fellow (refer to Appendix S2 and S3 [available in the online version only] for corresponding samples) • Evaluation of Fellow by Faculty Member (refer to Appendix S4 [available in the online version only] for a sample) • Multisource Evaluation of Fellow (peers, patients, self, and other professional staff members; refer to Appendix S5 [available in the online version only] for a sample) • Fellow Evaluation of Program (refer to Appendix S6 [available in the online version only] for a sample) • Faculty Evaluation of Program (refer to Appendix S7 [available in the online version only] for a sample) • Evaluation of Faculty Member by Fellow (refer to Appendix S8 [available in the online version only] for a sample) • ACGME Competency-Based Curriculum Goals and Objectives Sample for one educational experience at each level (Year 1 and Year 2; refer to Appendix S9 [available in the online version only] for a sample) • Specialty-Specific Application	Months 2-7
Step 4: Submitted New Fellowship Program Application to the CHLA GME Office • List of documents submitted to CHLA Graduate Medical Education Office ▪ CHLA GME New Program Approval Form ▪ Division Head Letter of Support, including a description of the funding source ▪ Block Diagram ▪ Rotation-Specific Goals and Objectives for all rotations ▪ Assessment of Impact on Existing Training Programs • Once all documents were submitted to the GME office, they scheduled the Program Director to present them at the next GME committee meeting.	Month 8
Step 5: Presented to CHLA GME Committee • The PD presented the proposed CI program to the GME Committee for approval to submit the application to the ACGME. • The committee voted to approve the program, allowing us to proceed with submitting the application to ACGME (voting took place on the same day). • Presentation Details: A brief 10- to 15-minute presentation with approximately five slides was given. Most questions focused on the block diagram, rotations, faculty involvement, allocated teaching time, and financial support.	Month 9
Step 6: DIO-Initiated Application on ADS • The DIO initiated the creation of an account on ADS for the ACGME CI Fellowship, entering basic information about the program, including the PD's details. • The PD then received an email with ADS account and login information. • The PD subsequently added the Program Coordinator and Associate PD to the ADS.	Month 9
Step 7: Prepared Application in ADS • The Program Coordinator entered and uploaded all necessary application information and attachments in ADS, which were reviewed and approved by the PD and Associate PD. • Completed three parts of the application in ADS ▪ Common Program Application ▪ Specialty-Specific Application ▪ Attachments (list of documents from Step 2) • Common Program Application includes ▪ Participating sites' information ▪ Faculty roster (core and non-core faculty) ▪ Faculty CV information ▪ Complement size (number of positions) ▪ Evaluation (competency, milestones) ▪ Duty hours, patient safety, and learning environment ▪ Trainee activity monitoring details ▪ Details on how the program ensures compliance with duty hour supervision and adherence to policies.	Month 9
Step 8: Submitted Application by DIO • The PD submitted the completed application to the DIO. • The DIO reviewed and submitted the final application to the ACGME. • Once the DIO submits the application in ADS, it cannot be altered or amended. • The DIO, PD, Associate PD, and Program Coordinator received a submission confirmation email from ADS.	Month 10
Step 9: Approval from the ACGME Review Committee • The application was reviewed by the ACGME Review Committee during their earliest prescheduled review dates. • The ACGME Review Committee determined that our program is in substantial compliance with the Program Requirements, granted Initial Accreditation, and communicated this decision through a Letter of Notification via ADS. • If the program is found not to be in substantial compliance, the status of Accreditation Withheld will be conferred, and the program must submit a new application if it wishes to continue pursuing accreditation. • It may take 4 to 12 months after submission of the application to undergo review and receive an accreditation decision from the Review Committee.	Months 11–13

Abbreviations: ACGME, Accreditation Council for Graduate Medical Education; ADS, Accreditation Data System; CHLA, Children's Hospital Los Angeles; DIO, designated institutional official; GME, Graduate Medical Education; PD, Program Director.

The timeline column reflects the approximate month-by-month progression from initial planning (Month 1) through final submission and review. While timelines may vary across institutions, this sequence illustrates the general process we followed.

## Fellowship Components

The training design of our CI Fellowship was informed by core ACGME requirements, institutional resources, and the varied backgrounds of incoming fellows. Below, we outline key components considered in our curriculum—highlighting which were included, which were deferred, and the rationale behind these decisions.

### Rotations

Fellows must undergo rigorous training in all foundational aspects of CI. We will accomplish that through rotations in various departments, including inpatient and outpatient settings, ancillary services, and research facilities. The aim is to provide fellows with practical, hands-on experience concerning processes, systems, and the role of CI in their implementation. For fellows with specific interests, unique elective opportunities are also available, such as specialized or advanced rotation topics, quality improvement projects, and additional research electives.

### Graduate-Level Certificate or Master's Degree

Fellows entering the CI program may have diverse backgrounds, including some with little to no prior experience in informatics. Because CI is an extensive field, not all institutions may have faculty who are specialized in every relevant area, nor may they have the time to develop a systematic curriculum. Consequently, a structured educational offering—like a Graduate Certificate or a Master's Degree in Clinical, Medical, Biomedical, or Health Informatics—may play an essential supplemental role for comprehensive training. Academic programs may offer a methodical exploration of all the key areas within CI and may serve as a valuable complement to the practical experience fellows gain during their institutional rotations.

ACGME does not mandate that fellows enroll in a graduate degree program. Some CI fellowships operate without requiring graduate-level courses, opting instead to develop in-house didactics and in-house educational content, 14 or to leverage online resources such as YouTube, Coursera, and webinar or seminar series. However, incorporating a graduate degree or a structured educational framework may enhance the overall learning experience of CI fellows. This comprehensive approach is particularly valuable given the challenges of delivering a full curriculum through faculty-led rotations alone. Additionally, it ensures that all the outcomes, competencies, and milestones specified by the ACGME for a CI fellowship are adequately covered. 5 8 15

However, at our institution, we opted not to include graduate coursework initially due to financial constraints. Tuition costs can be substantial and are difficult to sustain, especially without an internal informatics degree program. Relying on external university partnerships would further increase costs. Instead, we deliver curated didactic sessions supported by freely available educational resources. As our program matures, we plan to reassess this component and may pursue targeted funding to support graduate-level coursework for future cohorts based on evolving needs and feedback.

### Board Review Courses

After 2025, the CI Fellowship will be the only path to board certification. 16 17 Upon finishing an ACGME-accredited CI fellowship program, fellows are required to pass the CI subspecialty board exam to become board-certified 4 6 —that is, formally recognized by the American Board of Preventive Medicine (ABPM) or the American Board of Pathology (ABPath) as having demonstrated expertise in CI through a standardized national examination. The exam is held over a 2-week period, typically between October and November, 1 and consists of 200 multiple-choice questions. The exam is administered at various testing centers across the United States and several international locations. 1 The certification in CI is collaboratively administered by the ABPath and the ABPM. Both boards oversee the qualification of applicants, examination standards, and the design of the certificate. 1 The American Medical Informatics Association (AMIA) offers the CI Board Review Course (CIBRC), 18 which supplies review materials and educational instructions to assist CI fellows in successfully passing the CI subspecialty certification exam. The cost of this course varies depending on the chosen option: Online-only or the bundle option, which includes access to the live in-person meeting and the online course. When considering the bundle option, travel expenses should also be factored in addition to the course cost. More details related to course offerings, format, and pricing can be found on the CIBRC web site. 18 While enrolling in CIBRC is beneficial preparation, it is not a prerequisite or mandatory for taking the CI subspecialty board exam; it serves as an optional resource for fellows.

We recommend the AMIA CIBRC for our fellows, with current funding provided by the program. This choice reflects several factors: Its strong alignment with the board exam's structure and content, comprehensive topic coverage, and its role in easing faculty workload by complementing internal instruction. CIBRC is broadly regarded—by our CI board-certified faculty and external peers—as best practice for exam preparation.

### Electronic Health Record Physician Developer Training

The goal of the Physician Developer Training is to teach CI fellows how to tailor electronic health record (EHR) software to address unique workflow requirements in specialized and subspecialized medical disciplines, including customized documentation and ordering tools. The structure and content of these courses will differ depending on the institution and its corresponding EHR vendor(s). We advise program directors to reach out to the CI leadership at the institution and consult with EHR vendors for the best educational experiences. ACGME does not mandate fellows to enroll in EHR physician developer training; however, these programs can provide invaluable insights into the principles and inner workings of EHR systems as well as important skills that are needed in health systems.

At our institution, CI fellows participate in formal Oracle Health EHR (formerly Cerner Millennium) developer training, which is funded by the program. This training aligns with our primary EHR platform and equips fellows with foundational technical knowledge to support their informatics rotations. It also prepares them for hands-on, project-based work with EHR analysts on initiatives such as system optimization and clinical decision support.

### Clinical Informatics Fellowship Curriculum and Block Schedule

ACGME requires the submission of a detailed 2-year training curriculum and rotation plan. The plan includes a structured sequence of rotations and their locations (for programs that have more than one participating site), didactic sessions, biomedical informatics research, additional activities, and allocated vacation time. These components should optimize learning outcomes and professional success in CI, utilizing resources unique to the institution and, when applicable, affiliated hospitals for broader training experiences.

**Fig. 1:**
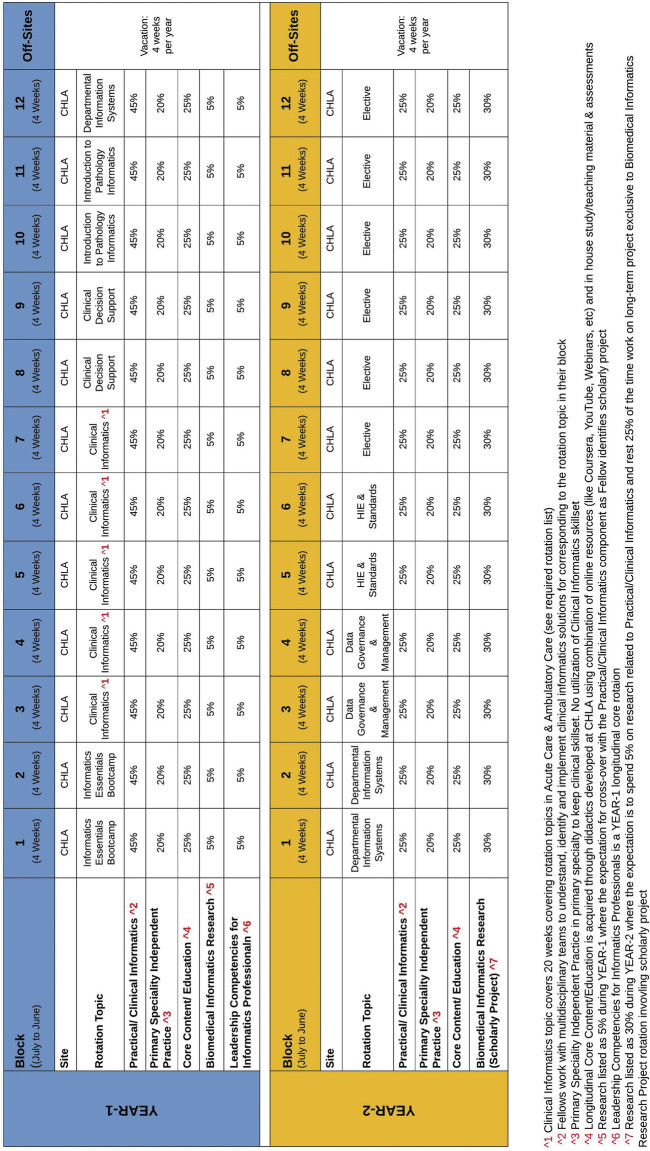
A block diagram detailing the curriculum and rotations for both years of the proposed CI fellowship program. CHLA, Children's Hospital Los Angeles; CI, clinical informatics.

To provide a high-level overview of our fellowship structure, Fig. 1 illustrates a visual roadmap outlining the distribution of core, elective, and longitudinal experiences throughout Year 1 and Year 2 of training. We organized our rotations into 4-week blocks. Rotations may have different lengths (e.g., one calendar month) dependent on the institutional requirements (see Fig. 1 ). Rotations include multidisciplinary collaboration to design and implement informatics solutions in the relevant area or domain that is the rotation's focus (e.g., a research informatics rotation might focus on designing a system to provide researchers with access to data). The curriculum includes mandatory core rotations and electives that fellows may choose based on their interests to gain deeper expertise (see Table 2 ). For instance, all our fellows undertake an “Introduction to Pathology Informatics” rotation, with an optional “Advanced Pathology Informatics” for those specializing in pathology seeking in-depth exposure.

**Table 2:** Proposed rotation topics and brief overviews for a Clinical Informatics Fellowship Program

Topic	Short overview
Core rotations	
Informatics Essentials Boot Camp	Introduces the Fellow to essential informatics tools and concepts, including computer programming, big-data analysis, Unix systems, data analytics, databases, and EHR Physician builder.
Clinical Informatics: Acute Care (Introductory)	Provides an introduction to informatics concepts specific to Inpatient, Critical Care, Emergency Department, and Anesthesia health care settings.
Clinical Informatics: Ambulatory Care (Introductory)	Introduces informatics concepts relevant to ambulatory, virtual care, and remote monitoring health care settings.
Clinical Decision Support	Offers the Fellow an opportunity to contribute to the development of a clinical decision support tool for use within the EHR.
Introduction to Pathology Informatics	Introduces informatics concepts, systems, and workflows specific to anatomic pathology, clinical pathology, clinical genomics, precision genomic medicine, and transfusion medicine.
Departmental Information Systems	Covers informatics concepts, systems, and workflows specific to radiology, pharmacy, and anesthesiology departments.
Data Governance, Management, and Acquisition	Introduces best practices for the governance, management, and acquisition of clinical and research data.
Biomedical Informatics Research Project	Provides faculty-guided opportunities for Fellows to apply their knowledge in a research project or join an ongoing faculty-initiated project.
Health Information Exchange and Standards	Introduces commonly used systems and standards that facilitate health care information exchange.
Leadership Competencies for Informatics Professionals	Develops an understanding of health care IT planning and implementation from a leadership perspective, focusing on organizational behavior, change management, collaboration, and communication.
Elective rotations	
Cybersecurity	Introduces concepts, systems, and workflows specific to cybersecurity.
Innovation and Digital Transformation	Focuses on health care innovation and digital transformation.
Clinical Informatics: Acute Care (Advanced)	Expands on the introductory rotation, training Fellows in advanced informatics concepts for Inpatient, Critical Care, Emergency Department, and Anesthesia settings.
Clinical Informatics: Ambulatory Care (Advanced)	Builds on the introductory rotation, offering advanced training in informatics for ambulatory, virtual care, and remote monitoring settings.
Advanced Pathology Informatics	Expands on the introductory rotation, providing advanced training in informatics for anatomic pathology, clinical pathology, clinical genomics, and transfusion medicine.
Clinical Bioinformatics and EHR Genomics	Requires prior completion of Introduction to Pathology Informatics; introduces advanced informatics concepts, systems, and workflows specific to clinical genome informatics, including the integration of genomic data into EHR, data exchange standards, and clinical decision support relevant to the implementation of precision genomic medicine practice and research.

Abbreviation: EHR, electronic health record.

Electives such as “Innovation and Digital Transformation” allow fellows to explore emerging topics in informatics. While there is flexibility in the specific topics of core and elective rotations, they generally reflect the institution's available expertise. Detailed descriptions, including rotation duration, location, objectives, and learning outcomes, are provided for each block (see Appendix S1 [available in the online version only] for samples). Although faculty assignments for each rotation might not be finalized at the application stage, they can be included if known.

In addition to scheduled rotations, CI fellows engage in a variety of activities to enhance their training. They maintain clinical skills through engaging in the independent practice of their core specialty. Core content and education are acquired longitudinally through didactics at CHLA, leveraging online resources like Coursera, YouTube, and webinars, along with in-house study materials. In our institution, during Year 1, fellows allocate 5% of their time to Biomedical Informatics Research, developing a scholarly project to be further explored in Year 2, with 30% dedicated effort. Leadership Competencies for Informatics Professionals is a longitudinal core rotation in Year 1 aimed at cultivating leadership skills. The specific distribution of efforts for these activities varies as institutions tailor them to their resources and needs. Some institutions add additional events such as fellow-organized journal clubs or monthly research presentations. Details of our CHLA rotation schedule and additional responsibilities are depicted in Fig. 1 .

### Dual Subspecialty Training Experience Opportunities with Clinical Informatics

Both certifying bodies for the subspecialty of CI offer unique opportunities to combine training in CI with another subspecialty. Typically, a CI fellowship requires 2 years of training. This time commitment may pose a challenge for individuals seeking to further specialize after completing a residency program. An “integrated training experience” (ITE), as offered by the ABPM, is a type of dual-training program that allows an individual fellow to complete training in two ACGME-accredited fellowships simultaneously by integrating the curricula and training experiences of the two individual fellowships. 19 ABPath describes similar types of integrated training (although not using the term, ITE) where a fellow may “simultaneously complete an additional one year of ACGME, ABPath subspecialty fellowship, during the same two years as the Clinical Informatics fellowship.” 20

This type of integrated training does not truncate or shorten training time or requirements for the CI fellowship. However, for example, since both fellowships may have research requirements, an ITE may allow the research conducted by a fellow to count toward both fellowships, thus reducing the total time needed to complete both fellowships.

As described by Palma et al., 20 proposals for dual training are typically tailored for each fellow individually and require advance approval from their respective informatics certifying board (ABPath for pathologists and ABPM for everyone else), as well as approval from the clinical specialty board, to ensure eligibility for both board examinations upon program completion. Various examples of ITE combinations have been explored, including CI fellowship training paired with Neuropathology, 21 Surgical Pathology, 19 Transfusion Medicine 19 (for fellows trained in Pathology), and with numerous other medical and pediatric subspecialties such as Critical Care Medicine 20 , Pediatric Infectious Diseases, 20 Maternal Fetal Medicine, 20 Hematology-Oncology, Neonatology, to name a few.

Key components of any petition for combined training include

1. A comprehensive plan ensuring the completion of all fellowship requirements for each specialty by the end of the combined training.
2. Demonstrated points of integration for rotational, didactic training, and scholarly activities, especially when requesting a reduction in the overall training duration.
3. Oversight and approval from both individual fellowship program directors and GME program leadership.

Detailed requirements can be found on the respective certifying board web sites. 21 22 Various models of integration have been demonstrated to fulfill the necessary requirements. 20

Common methods of integrating learning activities involve incorporating elective rotations, research initiatives, and other scholarly projects specifically designed to meet the requirements of both the clinical and CI fellowships. Ideally, an oversight committee comprises faculty from both disciplines that develops the integrated curriculum, typically using a block diagram approach, and continues to mentor the fellow throughout the process. ACGME Program Requirements for CI permit up to 20% of time per week (or 10 weeks of an academic year) for the “independent practice of their core specialty during their fellowship program.” 23 Integrating clinical practice time throughout the combined fellowship can be essential and ensures compliance with both fellowship programs' requirements. Several boards require regular annual progress reports to monitor the fellow's development during combined training, and successful completion of agreed-upon requirements by both program directors is mandatory for board eligibility.

Proposals demonstrating effective integration can often justify a reduction in the overall training duration, 20 particularly when traditional clinical fellowship training lasts for 2 or 3 years, and CI for 2 years. Because of the different rules of the ABPath, combining CI training with a 1-year pathology specialty fellowship can typically be accomplished in a total of 2 years. 21 This reduction in training time can result in cost savings for one or both programs due to lower fellow salary and benefit costs, which typically constitute a significant portion of any fellowship budget.

Effective planning and navigating logistical considerations are crucial to ensure the best learning experience for fellows in an ITE. The process often begins with recruitment, where identifying candidates who meet the qualifications for both the clinical and CI fellowship programs can be a challenge. Typically, although not always, a candidate applies to and matches with the clinical fellowship program and expresses an interest in formal CI training, either during the application process or shortly after matching. Timely initiation of the evaluation process for a candidate's eligibility for CI fellowship training, along with the submission of a proposal for a combined training experience to the boards, is essential. Demonstrating integration early in the process is vital, with most boards currently requiring submission of the application before any training begins.

By the end of 2025, ACGME-accredited fellowship training will be the sole pathway to board certification in CI by the ABPM 17 and ABPath. 21 Innovative solutions, such as dual fellowship training options, are crucial to attract candidates and to ensure a well-trained and qualified workforce, 9 and to prepare fellows for leadership roles that bridge their medical specialty and CI expertise.

During our program development, we proactively explored the feasibility of ITE to ensure readiness should a fellow express interest. Guidance from experienced CI program directors at established institutions helped us understand the logistical and curricular considerations of implementing this promising training model.

## Funding Needs and Mechanisms of Support for Clinical Informatics Fellowship Programs

Training a CI fellow involves a significant financial commitment, and many programs face challenges in securing sustainable funding. 9 While clinical programs usually allow attendings to shoulder larger clinical workloads and more billable revenue when supported by a fellow, CI does not have any billing codes, and while clinicians perform work that improves care delivery, there are no means to bill payers for the work provided. 9 24 25

The CI Fellowship Program has added financial needs that extend beyond merely providing salaries for fellows. These additional expenses may include items listed under the “Training Consideration” section above, including tuition for graduate-level education in CI—either a certificate or a master's program—as well as costs associated with professional conferences, board review courses, and EHR vendor training for physicians. Other costs include office space, equipment, and books. Funding is also required to support part of the salary for Program Directors, Associate Program Directors, and Program Coordinators.

Funding sources vary widely among institutions. While some institutions, like ours, rely solely on their CI Fellowship's home department, others draw from multiple sources including the fellow's primary medical specialty (in exchange for clinical work), hospital and health care system administration, GME offices, biomedical informatics departments, health IT departments, research grants and philanthropic contributions, and funding from other external organizations. 24 For a comprehensive survey and analysis of the structure and funding of CI fellowships, refer to Patel et al., 2024. 24 Overall, streamlining and planning funding sources is key to ensuring CI program administration and sustainability.

## Key Steps and Documents for Fellowship Application Submission

We followed several major steps during the process of securing ACGME accreditation at our institution, including establishing the CI Fellowship Steering Committee, identifying faculty, designing rotations, submitting the New Fellowship Program Application to the CHLA GME Office, obtaining approval from the CHLA GME Committee, and initiating, submitting, and gaining approval for the ACGME CI fellowship application. These steps are arranged in chronological order in Table 1 .

As part of the ACGME application, several key documents must be submitted, including the CI program evaluation forms, desired educational outcomes/competencies, and policies related to work hours, well-being, and supervision. Below is a list of essential documents, with references to examples in the Supplementary Appendices (available in the online version only). These samples are intended as general guidelines or suggestions and may evolve over time. Aspiring program directors are advised to consult the ACGME application manual for the most current requirements.

### Specialty-Specific Application

The application is specific to the CI specialty, which can be found in the “Application” section on the ACGME web site in the section of the specialty that hosts the CI fellowship at your institution.

### Block Diagram

A block diagram for each year of education in the program (see Fig. 1 and Table 2 ; and the “CI Fellowship Curriculum and Rotations” section above).

### Semiannual and Final Evaluations of Fellows

Blank copies of the forms used for semiannual evaluations of fellows with feedback options and the final evaluation form that certifies a fellow has acquired the necessary knowledge, skills, and behaviors for independent practice upon completion of the program. Examples are provided in Appendices S2 and S3 (available in the online version only).

### Evaluation of Fellows by Faculty Members

A sample of the form faculty members use to evaluate fellows. An example is provided in Appendix S4 (available in the online version only).

### Multisource Evaluation of Fellows

A sample of the multisource evaluation form used by non-faculty evaluators, such as peers, patients, the fellows themselves, and other professional staff members. An example is provided in Appendix S5 (available in the online version only).

### Forms Used for Resident/Fellow Evaluation of Program

A sample of the evaluation form used by fellows to assess the program. An example is provided in Appendix S6 (available in the online version only).

### Forms Used for Faculty Evaluation of Program

A sample of the form used by faculty members to evaluate the program. An example is provided in Appendix S7 (available in the online version only).

### Forms Used for Evaluation of Faculty Members

A sample of the form fellows use to evaluate individual faculty members. An example is provided in Appendix S8 (available in the online version only).

### Goals and Objectives

This document includes a sample of competency-based goals and objectives for one educational experience at each level of training (Year 1 and Year 2). The ACGME has identified core competencies (milestones) in the areas of patient care, medical knowledge, practice-based learning and improvement, systems-based practice, professionalism and communication, and interpersonal skills. These competencies form the foundation for curriculum design and assessments, and allow for monitoring of the fellows' progress throughout their training. An example is provided in Appendix S9 (available in the online version only).

### Program Letters of Agreement

All required Program Letters of Agreement (PLAs) for participating sites where fellows complete rotations. Since all rotations for our fellows will be conducted at CHLA, this requirement was not applicable for us. However, programs such as the University of Texas Southwestern Medical Center, where fellows rotate through the VA Hospital, Parkland Hospital, and Children’s Hospital in addition to the primary site of Clements University Hospital, were required to maintain three PLAs.

### Policy for Clinical and Educational Work Hours

The program's policies and procedures regarding clinical and educational work hours for fellows, including moonlighting policies, have been modeled closely after those used by other fellowships within our department and institution and were deemed acceptable by ACGME. We recommend that others consider adopting similar approaches.

### Policy for Supervision of Fellows

The policy detailing the supervision of fellows, covering patient care responsibilities, progressive management responsibilities, and the supervisory roles of faculty members, has been modeled closely after those used by other fellowships within our department and institution, and was deemed acceptable by ACGME. We recommend that others consider adopting similar approaches.

### Policy for Fellow and Faculty Member Well-Being

The policies addressing the well-being of fellows and faculty members have been modeled closely after those used by other fellowships within our department and institution and were deemed acceptable by ACGME. We recommend that others consider adopting a similar approach.

## Conclusion

This manuscript outlines our structured approach to designing, planning, and launching an ACGME-accredited CI fellowship. Through early stakeholder engagement, strategic curriculum development, and alignment with accreditation requirements, we established a program that reflects both institutional strengths and national training standards. By documenting our planning process, implementation framework, and curricular components, we hope to provide a practical model for institutions seeking to build or enhance their own CI fellowship programs.

## Lessons Learned and Future Directions

Our institutional journey toward the successful development and accreditation of an ACGME CI fellowship revealed several key insights and growth opportunities. A major contributor to our success was the early formation of a multidisciplinary steering committee that included CI board-certified faculty, GME representatives, and key enterprise informatics stakeholders, including C-suite CI leaders. Equally critical was the unwavering support, enthusiasm, and strategic guidance of our department chair, particularly in securing financial resources and navigating complex challenges. This early alignment of vision, leadership, and logistics significantly accelerated program development and the application process.

Among the greatest challenges were coordinating cross-departmental rotations, balancing core and elective experiences, and aligning training opportunities with both ACGME requirements and fellow interests. In response, we refined our scheduling strategies and expanded faculty engagement to ensure coverage and consistency across rotations.

Securing financial support emerged as another critical hurdle. Beyond covering fellow salaries, CI fellowships often require funding for board review courses, conference travel, graduate coursework, and EHR vendor training. Early and sustained engagement with departmental and institutional leadership was essential. Equally valuable was the guidance of external CI fellowship directors, who provided practical insights into funding models and shared lessons learned from their own programs.

While initial institutional support enabled a successful launch, long-term sustainability will require diversified funding sources—such as departmental cost-sharing, educational grants, philanthropic contributions, and GME support. We also devoted substantial time to evaluating the role, cost, and curricular fit of graduate-level coursework, a process that involved detailed discussions with institutional leaders.

Looking forward, we plan to strengthen training in emerging domains such as AI-driven clinical decision support, real-world data research, and digital transformation. 26 27 28 We will also continue to reassess the role of formal graduate coursework, weighing its educational value against financial feasibility as our program matures.

By incorporating these lessons, we aim to continuously improve our fellowship and contribute to a national model for high-impact, interdisciplinary informatics education—equipping our CI fellows with the skills and experience needed to lead effectively in the rapidly evolving landscape of health care informatics.

## Clinical Relevance Statement

The successful ACGME accreditation of the CI fellowship program signifies the establishment of a standardized training pathway that prepares physicians with specialized skills to enhance health care delivery and improve patient outcomes through the effective use of technology and data. This accredited pathway enables practitioners to develop expertise in CI, fostering innovation in health care systems. Patients benefit from improved care coordination, safety, and quality driven by the implementation of advanced informatics solutions.

## Multiple-Choice Questions

1. What is the key advantage of obtaining ACGME accreditation for a CI fellowship program?
  - It allows fellows to bypass board certification exams.
  - It ensures standardization of training and competency development.
  - It eliminates the need for faculty with informatics expertise.
  - It guarantees funding from external grants.
  **Correct Answer** : The correct answer is option b. It ensures standardization of training and competency development. ACGME accreditation sets rigorous educational standards for training programs, ensuring that fellows acquire the necessary competencies in CI. This standardization helps produce a well-qualified workforce while maintaining consistency across training institutions.
2. Which of the following was a critical step in the institution's journey toward ACGME accreditation for the CI fellowship?
  - Avoiding external collaboration to maintain institutional independence.
  - Establishing a multidisciplinary steering committee with institutional leaders.
  - Focusing only on clinical rotations without integrating informatics-specific training.
  - Submitting an application without detailed documentation.
  **Correct Answer** : The correct answer is option b. Establishing a multidisciplinary steering committee with institutional leaders. The manuscript emphasizes the importance of forming a diverse steering committee comprising institutional leaders, faculty, and informatics experts. This committee played a crucial role in planning, securing funding, and navigating the accreditation process.
3. Why is faculty involvement across multiple medical informatics specialties important for a CI fellowship?
  - It helps fulfill ACGME requirements by demonstrating broad expertise.
  - It allows fellows to focus only on one specific informatics application.
  - It eliminates the need for structured rotations.
  - It reduces the need for external expert guidance.
  **Correct Answer** : The correct answer is option a. It helps fulfill ACGME requirements by demonstrating broad expertise. CI is an interdisciplinary field, and a well-rounded program must include faculty with expertise in diverse informatics domains. This ensures comprehensive training that aligns with ACGME competencies and prepares fellows for various career paths.
4. What is a potential challenge faced by CI fellowship programs regarding funding?
  - Fellows can generate significant billable revenue through patient care.
  - Informatics work does not have designated billing codes like clinical specialties.
  - ACGME automatically provides funding for accredited programs.
  - External grants fully cover the cost of running the program.
  **Correct Answer** : The correct answer is option b. Informatics work does not have designated billing codes like clinical specialties. Unlike traditional medical specialties where clinical work generates billable revenue, CI lacks direct billing codes. This creates funding challenges, often requiring programs to secure financial support from multiple institutional sources.
