# Supplementary material for: Achieving ACGME Clinical Informatics Fellowship Accreditation: A Chronicle of Our Institution's Journey
Source: Appl Clin Inform. 2025 Sep 26;16(4):1171–83. doi: 10.1055/a-2680-5920 (PMC12473513; doi:10.1055/a-2680-5920)
Supplement: Supplementary file 1 — Supplementary Material [file 10-1055-a-2680-5920_27101434.pdf]

Thieme

Supplementary Appendixes

Achieving ACGME Clinical Informatics Fellowship Accreditation: A Chronicle of Our Institution's Journey

Table of Contents

APPENDIX S1 – ROTATION SYLLABI SAMPLES FOR ACUTE CARE 2

APPENDIX S2 –CI FELLOWSHIP SEMI-ANNUAL FELLOW EVALUATION *(SAMPLE)* 4

APPENDIX S3 – CI FELLOWSHIP FINAL FELLOW EVALUATION *(SAMPLE)*..........13

APPENDIX S4 – EVALUATION OF FELLOW BY FACULTY *(SAMPLE)* 16

APPENDIX S5 – MULTI-SOURCE EVALUATION OF FELLOW *(SAMPLE)* 17

APPENDIX S6 – FELLOW EVALUATION OF PROGRAM *(SAMPLE)* 19

APPENDIX S7 – FACULTY EVALUATION OF PROGRAM *(SAMPLE)* 22

APPENDIX S8 –EVALUATION OF FACULTY BY FELLOW *(SAMPLE)* 25

APPENDIX S9 – ACGME COMPETENCY BASED CURRICULUM: GOALS AND OBJECTIVES *(SAMPLE)* 28

Applied Clinical Informatics | DOI 10.1055/a-2680-5920 | © The Author(s).

Appendix S1 – Rotation Syllabi Samples for Acute Care

CLINICAL INFORMATICS – ACUTE CARE – INTRODUCTORY (CORE ROTATION)

ROTATION DURATION: 8 to 12 weeks (to be determined by Program Director and Fellow)
ROTATION LOCATION: Children's Hospital Los Angeles

ROTATION DESCRIPTION

This rotation will introduce the Fellow to informatics concepts specific to Inpatient, Critical Care, Emergency Department (ED), and Anesthesia healthcare settings. These setting presents a variety of unique Clinical Informatics challenges. For inpatient settings the key challenges are the care transitions potentially complex patients make and the non-linearity of inpatient care. While for ED and other acute care settings key challenges are the non-scheduled nature of patient arrival and the wide variability in patient acuity seen in these settings demand well designed systems to track, assess and display clinical data, and structure the process of care. The Fellow will gain first-hand exposure to (1) understand unique characteristics / challenges of Inpatient, Critical Care, ED, and Anesthesia healthcare workflows (2) use, implementation, and support of the EHR modules for these workflows, (3) decision-making process from an organization-wide committee level, (4) support clinical pathways and other algorithms to provide evidence-based support, (5) use of analytics to display and interpret trends for clinicians and administrators.

LEARNING OBJECTIVES

1. Illustrate the safety risks and the medication use process for patient care.
2. Examine the challenges of designing CDS for patient care; consider the uses of various time, patient, and provider triggers that can be sued to address these challenges.
3. Review the patient Medication Reconciliation Processes, conceptual challenges and those introduced by the EHR.
4. Explain how the concept of the patient record has evolved, how documentation practices have adapted and where they still need to change.
5. Outline the process of tracking, assessment, and structure of care, including use of standardized triage systems and the pros and cons of structured versus unstructured data in data collection.
6. Explore the systems used to provide decision support in a patient care setting and the optimal design of clinical pathways to provide evidence-based care, support educational needs, and encourage shared understanding of goals across the treatment team.
7. Review methods to display and analyze trends in a healthcare setting to support process improvement and planning of resources.

CLINICAL INFORMATICS – ACUTE CARE – ADVANCED

Applied Clinical Informatics | DOI 10.1055/a-2680-5920 | © The Author(s).

(ELECTIVE ROTATION)

ROTATION DURATION: 4 to 8 weeks (to be determined by Program Director and Fellow)
ROTATION LOCATION: Children's Hospital Los Angeles

ROTATION DESCRIPTION

It is expected that Fellow successfully complete Clinical Informatics Acute Care (Introductory) rotation prior to participating in this advanced rotation. This rotation will introduce the Fellow to advanced informatics concepts specific to Inpatient, Critical Care, Emergency Department (ED), and Anesthesia healthcare settings. The Fellow will gain in-depth knowledge to (1) understand unique characteristics / challenges of Inpatient, Critical Care, ED, and Anesthesia healthcare workflows (2) design concepts, use, implementation, and support of the EHR modules for these workflows, (3) advanced decision-making process from an organization-wide committee level, (4) implement clinical pathways and other algorithms to provide evidence-based support, (5) build analytics to display and interpret trends for clinicians and administrators.

LEARNING OBJECTIVES

1. Improve the safety risks and the medication use process for patient care.
2. Elaborate the challenges of designing CDS for patient care; consider the uses of various time, patient, and provider triggers that can be sued to address these challenges.
3. Assess the patient Medication Reconciliation Processes, conceptual challenges and those introduced by the EHR.
4. Elaborate how the concept of the patient record has evolved, how documentation practices have adapted and where they still need to change.
5. Build the process of tracking, assessment, and structure of care, including use of standardized triage systems and the pros and cons of structured versus unstructured data in data collection.
6. Plan the systems used to provide decision support in a patient care setting and the optimal design of clinical pathways to provide evidence-based care, support educational needs, and encourage shared understanding of goals across the treatment team.
7. Build methods to display and analyze trends in a healthcare setting to support process improvement and planning of resources.

Applied Clinical Informatics | DOI 10.1055/a-2680-5920 | © The Author(s).

Appendix S2 –CI Fellowship Semi-annual Fellow Evaluation *(sample)*

**Note – Adapted from the template provided by Dr. Christoph U. Lehmann, University of Texas Southwestern Medical Center's Clinical Informatics Program.

[Fellow Name]
[Fellow Program]
[Evaluation Period]

Evaluator
[Evaluator Committee Name]
Clinical Informatics

This formative evaluation section will help to monitor fellow learning and provide ongoing feedback that can be used by fellows to improve their learning in the context of provision of patient care or other educational opportunities. It will help:

- fellows identify their strengths and weaknesses and target areas that need work
- program directors and faculty members recognize where fellows are struggling and address problems immediately

Fellow's progress:

Fellow's strengths:

Applied Clinical Informatics | DOI 10.1055/a-2680-5920 | © The Author(s).

Fellow's weaknesses:

Recommendations to the Fellow:

Recommendations to the program director:

This summative evaluation evaluates a fellow's learning by comparing the fellows against the goals and objectives of the rotation and program, respectively.

Applied Clinical Informatics | DOI 10.1055/a-2680-5920 | © The Author(s).

Clinical Informatics Fellow Clinical Competence Evaluation

Check "N/A" whenever one's personal observations are insufficient for reliable evaluation of the fellow's attributes, knowledge, or performance. Comments are required for "marginal" or "unsatisfactory" ratings.

PROFESSIONALISM

Demonstrates a commitment to carrying out professional responsibilities; adherence to ethical principles; and sensitivity and responsiveness to the needs of a diverse patient population. Characteristics include respect, compassion, honesty, integrity, reliability, accountability, and commitment to professional development.

○	○	○	○	○	○
Rarely Meets Expectations	Sometimes Meets	Meets Expectations	Sometimes Exceeds	Consistently Exceeds	N/A

Dependably completes assigned tasks in a timely manner; assists team members when requested; respects assigned schedules. Anticipates team needs and assists as needed. Anticipates team needs and takes leadership role to independently implement solutions.

○	○	○	○	○	○
Rarely Meets Expectations	Sometimes Meets	Meets Expectations	Sometimes Exceeds	Consistently Exceeds	N/A

Demonstrates cultural competency; recognizes cultural differences that may affect clinical care, diagnoses, or the design/implementation of clinical systems, and identifies and avoids biases.

○	○	○	○	○	○
Rarely Meets Expectations	Sometimes Meets	Meets Expectations	Sometimes Exceeds	Consistently Exceeds	N/A

Acknowledges personal errors and puts the interests of patients and their families first; engages in ethical behavior. Models appropriate professional conduct without external guidance. Recognizes lapses in professionalism and provides assistance to other team members and colleagues.

○	○	○	○	○	○
Rarely Meets Expectations	Sometimes Meets	Meets Expectations	Sometimes Exceeds	Consistently Exceeds	N/A

Applied Clinical Informatics | DOI 10.1055/a-2680-5920 | © The Author(s).

INTERPERSONAL AND COMMUNICATION SKILLS

Consistently and actively engages in collaborative communication with all members of the team. Demonstrates effective communication strategies that enable collaborative work.

○	○	○	○	○	○
Rarely Meets Expectations	Sometimes Meets	Meets Expectations	Sometimes Exceeds	Consistently Exceeds	N/A

Demonstrates effective strategies that enable effective communication with patients and families. Translates communication skills into the design and development of clinical information systems. Participates in the implementation/maintenance of information systems to facilitate communication between providers and patients, families, and the public.

○	○	○	○	○	○
Rarely Meets Expectations	Sometimes Meets	Meets Expectations	Sometimes Exceeds	Consistently Exceeds	N/A

SYSTEMS-BASED PRACTICE

Demonstrates knowledge of electronic and related healthcare systems, works efficiently, and utilizes system resources to provide cost-effective informatics solutions.

○	○	○	○	○	○
Rarely Meets Expectations	Sometimes Meets	Meets Expectations	Sometimes Exceeds	Consistently Exceeds	N/A

Demonstrates knowledge of the etiology of negative consequences of clinical information systems. Identifies potential unintended consequences of information systems and process implementation.

○	○	○	○	○	○
Rarely Meets Expectations	Sometimes Meets	Meets Expectations	Sometimes Exceeds	Consistently Exceeds	N/A

Demonstrates basic knowledge of contracting and negotiation. Describes contracting and negotiation related to health IT purchases and consulting.

Applied Clinical Informatics | DOI 10.1055/a-2680-5920 | © The Author(s).

○	○	○	○	○	○
Rarely Meets Expectations	Sometimes Meets	Meets Expectations	Sometimes Exceeds	Consistently Exceeds	N/A

Demonstrates knowledge of the basics of practice finance. Describes how clinical information systems can support practice finance.

○	○	○	○	○	○
Rarely Meets Expectations	Sometimes Meets	Meets Expectations	Sometimes Exceeds	Consistently Exceeds	N/A

Recognizes and describes the principles of data warehousing and analysis. Performs queries of clinical data warehouses or repositories and interprets the results.

○	○	○	○	○	○
Rarely Meets Expectations	Sometimes Meets	Meets Expectations	Sometimes Exceeds	Consistently Exceeds	N/A

PRACTICE-BASED LEARNING AND IMPROVEMENT

Improves knowledge, skills and practice performance by analyzing and reflecting upon personal practice outcomes as well as appraising and applying scientific evidence to clinical informatics.

○	○	○	○	○	○
Rarely Meets Expectations	Sometimes Meets	Meets Expectations	Sometimes Exceeds	Consistently Exceeds	N/A

Recognizes limits of own knowledge. Reflects upon errors in a group setting.

○	○	○	○	○	○
Rarely Meets Expectations	Sometimes Meets	Meets Expectations	Sometimes Exceeds	Consistently Exceeds	N/A

Describes variations of errors and the broader impact on patient care. Participates in implementing a solution to resolve information system errors that impact patient care.

Applied Clinical Informatics | DOI 10.1055/a-2680-5920 | © The Author(s).

O	O	O	O	O	O
Rarely Meets Expectations	Sometimes Meets	Meets Expectations	Sometimes Exceeds	Consistently Exceeds	N/A

Applies evidence-based medicine in presentations, such as at journal club and, as appropriate, identifies research project early in fellowship. Applies evidence-based medicine principles in presentations at national or international meetings and/or prepares and submits articles to peer-reviewed publications.

O	O	O	O	O	O
Rarely Meets Expectations	Sometimes Meets	Meets Expectations	Sometimes Exceeds	Consistently Exceeds	N/A

MEDICAL KNOWLEDGE

Applies basic medical and clinical knowledge in Clinical Informatics related patient care. Approaches clinical situations with timely investigation, analysis, and planning.

O	O	O	O	O	O
Rarely Meets Expectations	Sometimes Meets	Meets Expectations	Sometimes Exceeds	Consistently Exceeds	N/A

Demonstrates basic knowledge of enterprise-wide system architecture and integration.

O	O	O	O	O	O
Rarely Meets Expectations	Sometimes Meets	Meets Expectations	Sometimes Exceeds	Consistently Exceeds	N/A

Demonstrates basic knowledge of programming, databases, and interface standards.

O	O	O	O	O	O
Rarely Meets Expectations	Sometimes Meets	Meets Expectations	Sometimes Exceeds	Consistently Exceeds	N/A

Applied Clinical Informatics | DOI 10.1055/a-2680-5920 | © The Author(s).

Demonstrates skills for effective leadership. Recognizes the elements of effective management (e.g., conflict resolution).

○	○	○	○	○	○
Rarely Meets Expectations	Sometimes Meets	Meets Expectations	Sometimes Exceeds	Consistently Exceeds	N/A

Assesses organizational culture and readiness for change. Develops effective strategies for promoting adoption/optimization of clinical information systems.

○	○	○	○	○	○
Rarely Meets Expectations	Sometimes Meets	Meets Expectations	Sometimes Exceeds	Consistently Exceeds	N/A

PATIENT CARE

Recognizes the need for a process in implementing new technology.

○	○	○	○	○	○
Rarely Meets Expectations	Sometimes Meets	Meets Expectations	Sometimes Exceeds	Consistently Exceeds	N/A

Describes the cost-benefit analysis process for new technology.

○	○	○	○	○	○
Rarely Meets Expectations	Sometimes Meets	Meets Expectations	Sometimes Exceeds	Consistently Exceeds	N/A

Identifies the principles of decision science as related to clinical decision making.

○	○	○	○	○	○
Rarely Meets Expectations	Sometimes Meets	Meets Expectations	Sometimes Exceeds	Consistently Exceeds	N/A

Applied Clinical Informatics | DOI 10.1055/a-2680-5920 | © The Author(s).

Evaluates clinical informatics systems used in documentation and coordination of patient care (e.g., usability, effectiveness)

○	○	○	○	○	○
Rarely Meets Expectations	Sometimes Meets	Meets Expectations	Sometimes Exceeds	Consistently Exceeds	N/A

Manages project expectations of end-users and leadership while balancing competing priorities. Monitors project scope and prevents or addresses uncontrolled changes or continuous growth in project scope.

○	○	○	○	○	○
Rarely Meets Expectations	Sometimes Meets	Meets Expectations	Sometimes Exceeds	Consistently Exceeds	N/A

Participates in a system evaluation and the assessment of need for new systems.

○	○	○	○	○	○
Rarely Meets Expectations	Sometimes Meets	Meets Expectations	Sometimes Exceeds	Consistently Exceeds	N/A

Participates in the development of a requirements document. Applies usability frameworks and human interface design standards.

○	○	○	○	○	○
Rarely Meets Expectations	Sometimes Meets	Meets Expectations	Sometimes Exceeds	Consistently Exceeds	N/A

Summary Evaluation:

The Fellow is ready to proceed to the next level of training

[ ] Yes

[ ] No

There is a need for remedial education

Applied Clinical Informatics | DOI 10.1055/a-2680-5920 | © The Author(s).

[ ] Yes

[ ] No

Applied Clinical Informatics | DOI 10.1055/a-2680-5920 | © The Author(s).

Thieme

Appendix S3 – CI Fellowship Final Fellow Evaluation *(sample)*

**Note – Adapted from the template provided by Dr. Christoph U. Lehmann, University of Texas Southwestern Medical Center's Clinical Informatics Program.

Final Fellow Evaluation

[Fellow Name]
[Fellow Program]
[Evaluation Period]

Evaluator
[Evaluator Committee Name]
Clinical Informatics

This formative evaluation section will evaluate the Fellow's learning and knowledge acquisition in Clinical Informatics over the course of the training. It is to be completed at the end of the Fellow's training.

Prior to completing the next section, please complete the milestone based **Clinical Informatics Fellow Clinical Competence Evaluation** below.

Fellow's progress:

Fellow's strengths:

Applied Clinical Informatics | DOI 10.1055/a-2680-5920 | © The Author(s).

Fellow’s weaknesses:

Recommendations to the Fellow:

Recommendations to the program director:

Summary Evaluation:

The Fellow needs remedial education or repetition of rotations

[ ] Yes

[ ] No

The fellow has demonstrated the knowledge, skills, and behaviors to enter autonomous practice of Clinical Informatics.

[ ] Yes

[ ] No

Applied Clinical Informatics | DOI 10.1055/a-2680-5920 | © The Author(s).

The following evaluation evaluates a fellow's learning by comparing the fellows against the Clinical Informatics Milestones.

The Clinical Informatics Fellow Clinical Competence Evaluation form for the final year is the same as the one listed above in **Appendix S2 – CI Fellowship Semi-Annual Fellow Evaluation.**

Appendix S4 – Evaluation of Fellow by Faculty *(sample)*

The Evaluation of Fellow by Faculty form content is the same as the one listed above in **Appendix S2 – CI Fellowship Semi-Annual Fellow Evaluation.**

Applied Clinical Informatics | DOI 10.1055/a-2680-5920 | © The Author(s).

Appendix S5 – Multi-Source Evaluation of Fellow *(sample)*

Fellow:	**[First Name] [Last Name]**
Evaluation Period:	**[99/99/9999] to [99/99/9999]**

Evaluator:	**[First Name] [Last Name]**
Rotation Name:	**[Rotation Name]**

Personal and Professional Development

The fellow works effectively with others as a member or leader of a health care team or another professional group.

Strongly Disagree	Disagree	Neither Disagree nor Agree	Agree	Strongly Agree
○ 1	○ 2	○ 3	○ 4	○ 5

○ **Not Applicable**

Demonstrates a commitment to carrying out professional responsibilities, and adherence to ethical principles.

Strongly Disagree	Disagree	Neither Disagree nor Agree	Agree	Strongly Agree
○ 1	○ 2	○ 3	○ 4	○ 5

○ **Not Applicable**

Communicates well; is caring and respectful with patients and families.

Strongly Disagree	Disagree	Neither Disagree nor Agree	Agree	Strongly Agree
○ 1	○ 2	○ 3	○ 4	○ 5

○ **Not Applicable**

Demonstrates appropriate skills for obtaining and using scientific information to enhance patient care and self-improvement.

Strongly Disagree	Disagree	Neither Disagree nor Agree	Agree	Strongly Agree
○ 1	○ 2	○ 3	○ 4	○ 5

○ **Not Applicable**

Shows respect to co-workers and provides information when needed.

Strongly Disagree	Disagree	Neither Disagree nor Agree	Agree	Strongly Agree
○ 1	○ 2	○ 3	○ 4	○ 5

○ **Not Applicable**

Demonstrates interpersonal and communication skills that result in effective information exchange and collaboration with all team members.

Strongly Disagree	Disagree	Neither Disagree nor Agree	Agree	Strongly Agree
○ 1	○ 2	○ 3	○ 4	○ 5

○ **Not Applicable**

Receives constructive verbal and/or written feedback in a professional manner.

Strongly Disagree	Disagree	Neither Disagree nor Agree	Agree	Strongly Agree
○ 1	○ 2	○ 3	○ 4	○ 5

○ **Not Applicable**

Applied Clinical Informatics | DOI 10.1055/a-2680-5920 | © The Author(s).

Recognizes and accepts his/her own limitations and incorporates feedback into improvement activities.

Strongly Disagree	Disagree	Neither Disagree nor Agree	Agree	Strongly Agree
○ 1	○ 2	○ 3	○ 4	○ 5

○ Not Applicable

Comments ***(Please write about Strengths, Weaknesses and Areas for Improvement)***

*** * Confidential Comments** ***(The following comments will only be seen by the Program Director)***

Applied Clinical Informatics | DOI 10.1055/a-2680-5920 | © The Author(s).

Appendix S6 – Fellow Evaluation of Program *(sample)*

Fellow: **[First Name] [Last Name]**
Evaluation Period: **[99/99/9999] to [99/99/9999]**

Evaluator: **[First Name] [Last Name]**
Rotation Name: **[Rotation Name]**

Assess the programs clinical environment in terms of patient safety, supervision, and support from Attendings and Faculty.

Comments and/or Observations:

Describe how your individual learning plan has guided you in self-directed learning.

Comments and/or Observations:

Explain how your fellowship program addresses your communication skills. For example, how have you learned to communicate with physicians and other health professionals?

Comments and/or Observations:

How have your faculty and program director set an example of professionalism? Describe what you have learned about professionalism from your faculty and program director.

Comments and/or Observations:

Describe how the fellowship program has or has not assisted you with development of your hypothesis, methods, and/or data analysis.

Comments and/or Observations:

Explain what you find useful about Grand Rounds, and what, if anything you would like to see incorporated.

Comments and/or Observations:

Applied Clinical Informatics | DOI 10.1055/a-2680-5920 | © The Author(s).

Explain what you find useful about Journal Club, and if there are any changes or additions you would like to see incorporated.

Comments and/or Observations:

Overall, I am treated fairly, with respect, and I am made to feel "part of the team"

- (Select one)
- N/A
- No
- Yes

Overall, the quality and amount of teaching by the faculty is:

- (Select one)
- Not Applicable
- Poor
- Below Expectations
- Meets Expectations
- Above Expectations
- Outstanding

Overall, the quality and amount of supervision by the faculty is:

- (Select one)
- Not Applicable
- Poor
- Below Expectations
- Meets Expectations
- Above Expectations
- Outstanding

The mix of clinical experiences I have had during this year are:

- (Select one)
- Not Applicable
- Poor
- Below Expectations
- Meets Expectations
- Above Expectations
- Outstanding

The use of program resources to enhance my learning experience is:

- (Select one)
- Not Applicable
- Poor
- Below Expectations
- Meets Expectations
- Above Expectations
- Outstanding

Please describe any other strengths or opportunities for growth in your program that have not already been covered in the questions above.

* * **Confidential Comments** *(The following comments will only be seen by the Program Director)*

Appendix S7 – Faculty Evaluation of Program *(sample)*

Fellow: **[First Name] [Last Name]**
Evaluation Period: **[99/99/9999] to [99/99/9999]**

Evaluator: **[First Name] [Last Name]**
Rotation Name: **[Rotation Name]**

Describe the strenghts of the fellowship program.

Comments and/or Observations:

What opportunities are available for the fellowship to improve?

Comments and/or Observations:

Do you find Division Grand Rounds to be a useful experience for the fellows?

Describe what you would like to see added and/or change for these lectures.

(Select one)
N/A
No
Yes

Comments and/or Observations:

Applied Clinical Informatics | DOI 10.1055/a-2680-5920 | © The Author(s).

Thieme

Do you find Journal Club to be a useful experience for the fellows?

Describe what you would like to see added and/or change for these lectures.

(Select one)
N/A
No
Yes

Comments and/or Observations:

Do you feel the program adequately prepares fellows for their career? Explain how the program could better prepare fellows if necessary.

(Select one)
N/A
No
Yes

Comments and/or Observations:

Were you a mentor and/or did you serve on a program committee?

If yes, please note whether you were a mentor, committee or both. Also, please describe your experience and any changes you would like to see.

(Select one)
N/A
No
Yes

Comments and/or Observations:

Were the goals and objectives of the fellowship program clear to you?

(Select one)
N/A
No
Yes

Comments and/or Observations:

Describe how you see your role as a faculty member within the fellowship program.

Comments and/or Observations:

Applied Clinical Informatics | DOI 10.1055/a-2680-5920 | © The Author(s).

What would you like to see added or changed about the fellowship program?

Comments and/or Observations:

Additional Comments

Applied Clinical Informatics | DOI 10.1055/a-2680-5920 | © The Author(s).

Appendix S8 –Evaluation of Faculty by Fellow *(sample)*

Fellow:	**[First Name] [Last Name]**
Evaluation Period:	**[99/99/9999] to [99/99/9999]**

Evaluator:	**[First Name] [Last Name]**
Rotation Name:	**[Rotation Name]**

Medical Knowledge

Demonstrated significant expertise in his/her field

Strongly Disagree	Disagree	Neither Disagree nor Agree	Agree	Strongly Agree
○ 1	○ 2	○ 3	○ 4	○ 5

○ **Not Applicable**

Interpersonal and Communication Skills

Communicated expectations and guidelines so that I knew what was expected of me

Strongly Disagree	Disagree	Neither Disagree nor Agree	Agree	Strongly Agree
○ 1	○ 2	○ 3	○ 4	○ 5

○ **Not Applicable**

Answered questions thoughtfully and precisely

Strongly Disagree	Disagree	Neither Disagree nor Agree	Agree	Strongly Agree
○ 1	○ 2	○ 3	○ 4	○ 5

○ **Not Applicable**

Practice-Based Learning and Improvement

Created a positive learning environment

Strongly Disagree	Disagree	Neither Disagree nor Agree	Agree	Strongly Agree
○ 1	○ 2	○ 3	○ 4	○ 5

○ **Not Applicable**

Professionalism

Demonstrated an interest in teaching

Strongly Disagree	Disagree	Neither Disagree nor Agree	Agree	Strongly Agree
○ 1	○ 2	○ 3	○ 4	○ 5

○ **Not Applicable**

Interacted with me from a position of mutual respect

Strongly Disagree	Disagree	Neither Disagree nor Agree	Agree	Strongly Agree
○ 1	○ 2	○ 3	○ 4	○ 5

○ **Not Applicable**

Served as an effective role model

Strongly Disagree	Disagree	Neither Disagree nor Agree	Agree	Strongly Agree
○ 1	○ 2	○ 3	○ 4	○ 5

○ Not Applicable

Supervision

Provided appropriate amount of time for my instruction

Strongly Disagree	Disagree	Neither Disagree nor Agree	Agree	Strongly Agree
○ 1	○ 2	○ 3	○ 4	○ 5

○ Not Applicable

Teaching Skills

Asked questions in non-threatening way

Strongly Disagree	Disagree	Neither Disagree nor Agree	Agree	Strongly Agree
○ 1	○ 2	○ 3	○ 4	○ 5

○ Not Applicable

Emphasized problem-solving i.e., thought processes leading to decisions

Strongly Disagree	Disagree	Neither Disagree nor Agree	Agree	Strongly Agree
○ 1	○ 2	○ 3	○ 4	○ 5

○ Not Applicable

Stimulated me to read, research and review pertinent topics

Strongly Disagree	Disagree	Neither Disagree nor Agree	Agree	Strongly Agree
○ 1	○ 2	○ 3	○ 4	○ 5

○ Not Applicable

Personal and Professional Development

Provides leadership that enhances team functioning, the learning environment and/or health care system/environment with the ultimate intent of improving care of patients.

Strongly Disagree	Disagree	Neither Disagree nor Agree	Agree	Strongly Agree
○ 1	○ 2	○ 3	○ 4	○ 5

○ Not Applicable

Responsibility

Provided feedback that that assisted me in improving my performance as a Clinical Informaticist

Strongly Disagree	Disagree	Neither Disagree nor Agree	Agree	Strongly Agree
○ 1	○ 2	○ 3	○ 4	○ 5

○ Not Applicable

Applied Clinical Informatics | DOI 10.1055/a-2680-5920 | © The Author(s).

Comments ***(Please write about Strengths, Weaknesses and Areas for Improvement)***

*** * Confidential Comments** ***(The following comments will only be seen by the Program Director)***

Applied Clinical Informatics | DOI 10.1055/a-2680-5920 | © The Author(s).

Appendix S9 – ACGME Competency Based Curriculum: Goals and Objectives *(sample)*

Note 1: The ACGME CI Fellowship application requires competency-based curriculum goals and objectives descriptions for one rotation topic in Year 1 and another in Year 2. In this example, we have chosen "Acute Care Introductory" for Year 1 and "Clinical Informatics – Acute Care Advanced" for Year 2 rotations.

Note 2: This document outlines the ACGME CI Fellowship Competency for "Patient Care and Procedure Skills" only. Similar documentation must be created for the other required competencies: "Medical Knowledge," "Practice-Based Learning and Improvement," "Interpersonal and Communication Skills," "Professionalism," and "Systems-Based Practice."

Note 3: The list of competencies and their requirements may vary over time; refer to the ACGME application manual for the most current information.

SAMPLE

CLINICAL INFORMATICS – ACUTE CARE INTRODUCTORY (YEAR 1) & CLINICAL INFORMATICS – ACUTE CARE ADVANCED (YEAR 2) ROTATIONS

(Refer to Attachments: Clinical Informatics Acute Care Introductory & Advanced Rotations Syllabi in Appendix 1)

**ACGME COMPETENCE: PATIENT CARE AND PROCEDURE SKILLS**<br><br>**Goal 1: To leverage acute care (Inpatient, Critical Care, Emergency Department, and Anesthesia) clinical informatics tools to support patient care in an academic pediatric hospital setting that is compassionate, appropriate, and effective for the treatment of health problems and the promotion of health.**		
**Fellows Objectives**	**Instructional Strategies**	**Assessment Method(s)**

Applied Clinical Informatics | DOI 10.1055/a-2680-5920 | © The Author(s).

1. Use informatics tools to improve assessment, interdisciplinary care planning, management, coordination, and follow-up of patients (YEAR 1, YEAR 2)	• Attend weekly Clinical Informatics Team Meetings **(Primary Instructional Strategy)**<br>• Complete required informatics readings, lectures, and assignments<br>• Develop and design a Clinical Informatics Improvement project for an acute care setting (Inpatient, Critical Care, Emergency Department, and Anesthesia)<br>• Meet on a regular basis with the preceptor to discuss readings and projects	• Direct observation<br>• “Fellow Performance Evaluation” by faculty for each rotation<br>• Self-Assessment and Reflection<br>• Project evaluation
2. Use informatics tools, such as electronic health records or personal health records, to facilitate the coordination and documentation of key events in patient care, such as family communication, consultation around goals of care, immunizations, advance directive completion, and involvement of multiple team members as appropriate (YEAR 1, YEAR 2)	• Attend weekly Clinical Informatics Team Meetings **(Primary Instructional Strategy)**<br>• Complete required informatics readings, lectures, and assignments<br>• Develop and design a Clinical Informatics Improvement project for an acute care setting (Inpatient, Critical Care, Emergency Department, and Anesthesia)<br>• Meet on a regular basis with the preceptor to discuss readings and projects	• Direct observation<br>• “Fellow Performance Evaluation” by faculty for each rotation<br>• Self-Assessment and Reflection<br>• Project evaluation
3. Demonstrate competence in the identification of changes needed in organizational processes and clinician practices to optimize health system operational effectiveness (YEAR 1, YEAR 2)	• Attend weekly Clinical Informatics Team Meetings **(Primary Instructional Strategy)**<br>• Complete required informatics readings, lectures, and assignments<br>• Develop and design a Clinical Informatics Improvement project for an acute care setting (Inpatient, Critical Care, Emergency Department, and Anesthesia)<br>• Meet on a regular basis with the preceptor to discuss readings and projects	• Direct observation<br>• “Fellow Performance Evaluation” by faculty for each rotation<br>• Self-Assessment and Reflection<br>• Project evaluation

Applied Clinical Informatics | DOI 10.1055/a-2680-5920 | © The Author(s).

4. Demonstrate competence in the analysis of patient care workflow and processes to identify information system features that will support improved quality, efficiency, effectiveness, and safety of clinical services (YEAR 1, YEAR 2)	• Attend weekly Clinical Informatics Team Meetings **(Primary Instructional Strategy)**<br>• Complete required informatics readings, lectures, and assignments<br>• Develop and design a Clinical Informatics Improvement project for an acute care setting (Inpatient, Critical Care, Emergency Department, and Anesthesia)<br>• Meet on a regular basis with the preceptor to discuss readings and projects	• Direct observation<br>• “Fellow Performance Evaluation” by faculty for each rotation<br>• Self-Assessment and Reflection<br>• Project evaluation
5. Combine an understanding of informatics concepts, methods, and health IT to develop, implement and refine clinical decision support systems (YEAR 1, YEAR 2)	• Attend weekly Clinical Informatics Team Meetings **(Primary Instructional Strategy)**<br>• Complete required informatics readings, lectures, and assignments<br>• Develop and design a Clinical Informatics Improvement project for an acute care setting (Inpatient, Critical Care, Emergency Department, and Anesthesia)<br>• Meet on a regular basis with the preceptor to discuss readings and projects	• Direct observation<br>• “Fellow Performance Evaluation” by faculty for each rotation<br>• Self-Assessment and Reflection<br>• Project evaluation
6. Evaluate the impact of information system implementation and use on patient care and users (YEAR 1, YEAR 2)	• Attend weekly Clinical Informatics Team Meetings **(Primary Instructional Strategy)**<br>• Complete required informatics readings, lectures, and assignments<br>• Develop and design a Clinical Informatics Improvement project for an acute care setting (Inpatient, Critical Care, Emergency Department, and Anesthesia)<br>• Meet on a regular basis with the preceptor to discuss readings and projects	• Direct observation<br>• “Fellow Performance Evaluation” by faculty for each rotation<br>• Self-Assessment and Reflection<br>• Project evaluation

Applied Clinical Informatics | DOI 10.1055/a-2680-5920 | © The Author(s).
